# Tin and Gold Combined as a Filling Material

**Published:** 1887-01

**Authors:** E. G. Betty


### Tin and Gold Combined as a Filling Material.

BY E. G. BETTY, D.D.S.

Tin and gold combined for the purpose of filling is not altogether a new thing, yet it is new to the great majority of practitioners, and it is for their benefit that the writer wishes to jot down a few facts and ideas lately gleaned, for there is nothing like striking while the iron's hot.

Dr. Allport's paper, read at the past meeting of the Ohio State Dental Society at Toledo, touched upon the subject, and that with Dr. Brophy's clinical demonstration provoked a thorough discussion of the matter, which was not only intensely interesting, but of great practical advantage to all present.

Some few years ago Dr. A. A. Blount, of Geneva, Switzerland (since returned to Springfield, Ohio), advocated the lining of cavities with a layer of tin foil pressed and burnished tightly against the walls of the cavity, leaving a cavity within to be filled with gold. If memory serves rightly, the gold might also be used as the lining and the tin for the filling, though the former was the method usually adopted.

In Dr. Blount's system the two metals were kept apart and distinct, though the tin in contact with the gold exerted what has since been denominated a "peculiar therapeutic effect," whatever that may be. Dr. Allport states in his paper that when tin and gold are combined, in contact, and in the mouth, the sulphide of tin is formed, and it is this which constitutes the preserving power of such a filling. He further states it is essential that the tin come in contact with the cervical or other borders of the cavity in order that the action be more complete and that the sulphide formed fills up any minute crevices or interstices which may result in the introduction of the filling. It was also stated by several that a filling of tin and gold combined crystallizes after a time and becomes very hard. This is indeed a peculiar and very valuable property, especially, when one considers the extreme softness of the two metals used. The hardness is so great as to perfectly resist the wear and tear of mastication upon the grinding surface, and would be equally so on the corners of proximal fillings in bicuspids and molars if the fillings were allowed to remain long enough for the change to take place. This one thing alone is of immense advantage and sufficient to commend the filling to the profession, and every one should at least make a few fillings to test it for himself under his own eyes, for that's the only way of converting the skeptical. The method of preparing the two metals is very simple. A sheet of No. 4 tin and No. 4 non-cohesive gold are laid upon one another, cut into two or three strips and rolled up loosely, then cut into suitable lengths, usually from one-half to an inch. Before introducing into the cavity the roll is warmed slightly in the spirit flame and packed into place with a hand pressure instrument. When the cavity is full and running over, burnish solidly, using the mallet. The writer, who has been using a great deal of Robinson's Textile Foil for the past six years, greatly prefers the mallet all the way through the operation, the condensation thus produced being as nearly absolute as it is possible to make it, consistent of course with the resisting power of the teeth operated upon. The "combination" may be used altogether to fill a cavity, or it may be used in part, just as textile foil is, viz.: at the cervical border of a proximal cavity in a bicuspid or molar for instance; it may be packed in very solidly, filling from one-third to one-half of the cavity with it, the balance being built of any weight of cohesive gold foil, preferably, No. 60. The adaptation of the combined metals is exceedingly good, and furthermore, much time may be saved, thus relieving the strain upon both operator and patient, the latter being a point all will appreciate.

During a visit the writer made to Chicago in the early part of November, he had the pleasure of witnessing Dr. Harlan make a number of these fillings, and was fully impressed with the advantages to be derived. Dr. Harlan and others, of Chicago, all say that the darkening resulting from the combination does not permeate the tooth, in fact is advantageous, as it evidences the hardening or crystallizing mentioned above. Besides, the filling being introduced and finished in a much shorter space of time than with cohesive gold foil, it can be made with less cost to the patient and increases the revenue of the operator, in as much as he will have more of them to make, and not risk the failures incident upon the use of amalgam.

All those who have been using Robinson's metal will very readily fall into the way of using the combined tin and gold as the introduction of the latter is very much like that of the former, and also permits the use of a matrix in suitable cases. The thin tin matrix suggested by Dr. D. R. Jennings, of Cleveland, answers the purpose exceedingly well, as it is thin and will yield more or less, thus allowing a surplus of material to pass over the borders, and besides, the reflection of light from its surface greatly assists in illuminating the cavity.

Dr. Miller, of Berlin, has been investigating the effects of the combination upon the dentine and micro-organisms found in cavities, but with that, more or less all are familiar, so that repetition is unnecessary. Many little items of detail will suggest themselves to the individual, and may be made use of with advantage, but which are omitted here so that any one else may have a chance to study up the matter and make it the subject for a paper to one of the journals (REGISTER, preferred) or to his Society, in which event the writer will have accomplished part of his purpose in inditing these few lines.

